# Co-morbid depression is associated with poor work outcomes in persons with cardiovascular disease (CVD): A large, nationally representative survey in the Australian population

**DOI:** 10.1186/1471-2458-12-47

**Published:** 2012-01-18

**Authors:** Adrienne O'Neil, Emily D Williams, Christopher E Stevenson, Brian Oldenburg, Kristy Sanderson

**Affiliations:** 1 Department of Epidemiology and Preventive Medicine, School of Public Health and Preventive Medicine, Monash University, 89 Commercial Road, Melbourne, Victotia, Australia; 2 Menzies Research Institute Tasmania, University of Tasmania, Private Bag 23, Hobart, Tasmania, Australia

## Abstract

**Background:**

Co-morbid major depressive disorder (MDD) and cardiovascular disease (CVD) is associated with poor clinical and psychological outcomes. However, the full extent of the burden of, and interaction between, this co-morbidity on important vocational outcomes remains less clear, particularly at the population level. We examine the association of co-morbid MDD with work outcomes in persons with and without CVD.

**Methods:**

This study utilised cross-sectional, population-based data from the 2007 Australian National Survey of Mental Health and Wellbeing (n = 8841) to compare work outcomes of individuals with diagnostically-defined MDD and CVD, MDD but not CVD, CVD but not MDD, with a reference group of "healthy" Australians. Workforce participation was defined as being in full- or part-time employment. Work functioning was measured using a WHO Disability Assessment Schedule item. Absenteeism was assessed using the 'days out of role' item.

**Results:**

Of the four groups, those with co-morbid MDD and CVD were least likely to report workforce participation (adj OR:0.4, 95% CI: 0.3-0.6). Those with MDD only (adj OR:0.8, 95% CI:0.7-0.9) and CVD only (adj OR:0.8, 95% CI: 0.6-0.9) also reported significantly reduced odds of participation. Employed individuals with co-morbid MDD and CVD were 8 times as likely to experience impairments in work functioning (adj OR:8.1, 95% CI: 3.8- 17.3) compared with the reference group. MDD was associated with a four-fold increase in impaired functioning. Further, individuals with co-morbid MDD and CVD reported greatest likelihood of workplace absenteeism (adj. OR:3.0, 95% CI: 1.4-6.6). Simultaneous exposure to MDD and CVD conferred an even greater likelihood of poorer work functioning.

**Conclusions:**

Co-morbid MDD and CVD is associated with significantly poorer work outcomes. Specifically, the effects of these conditions on work functioning are synergistic. The development of specialised treatment programs for those with co-morbid MDD and CVD is required.

## Background

Depression and cardiovascular disease (CVD) are leading causes of health and economic burden globally [1]. By 2020, it is predicted that major depressive disorder (MDD) and coronary heart disease (CHD) will be the leading two global causes of disease burden [2]. A common medical co-morbidity, depression often co-exists with CVD. Depression can manifest before or after CVD onset leading to a range of poorer outcomes including decreased medication adherence, greater suicide risk [3], poorer health service utilisation, CHD risk factor profiles, survival [4] and some work outcomes [5]. Co-morbid mental and physical health conditions are highly prevalent in developed countries, such as the United States (US), United Kingdom (UK) and Australia [6], therefore the impact of co-morbid MDD and CVD on industry is likely to be great. However, to date, the burden of, and interaction between, this condition at the societal level remains unclear.

Poor health has been associated with both work absenteeism and presenteeism (attending work while sick). It is also the case that individuals with chronic conditions are less likely to be in full-time employment than those without [7]. Despite the benefits of active employment such as greater positive affect, less negative affect and fewer somatic complaints [8], people with a chronic condition are more likely to leave employment and retire early. Indeed, heart disease and depression have been reported as the two leading chronic diseases that contribute to labour force non-participation in developed countries [7]. The co-morbid burden of depression and other chronic conditions (e.g. musculoskeletal disorders [9]) on workforce participation has already been established; Baune (2007) demonstrated that MDD co-occurring with any medical disorder was strongly associated with lower full-time working status [10]. However, less is known about the specific burden of co-morbid MDD and CVD and how these two conditions interact to influence various aspects of working life. Indeed, when the impact of co-morbid MDD and a range of chronic conditions (including CHD) have been explored, co-morbid MDD has been shown to approximately double the likelihood of increased functional disability and work absence [11]. While elevated functional impairments may result from these conditions interacting rather than acting independently, evidence of such an effect remains inconsistent. Previous studies have shown a synergistic effect of co-morbid MDD and chronic physical conditions (e.g. diabetes) on functioning [12], but not on work absenteeism [11]. Further research is required to determine the nature of the relationship between MDD and CVD on key work outcomes.

The aim of the paper is to examine (i) the association of co-morbid MDD with work outcomes (workforce participation, work functioning and workplace absenteeism) in persons with and without CVD; and (ii) the way in which MDD and CVD interact to impact on work outcomes.

## Methods

### Study design and sampling

Cross-sectional, population-based data from the 2007 Australian National Survey of Mental Health and Wellbeing (NSMHWB) were utilised. This methodology has been described in detail elsewhere [13], but briefly, the sample was based on a stratified, multistage probability sample of persons aged 16-85 years living in private dwellings in Australia, excluding very remote areas. The overall response rate was 60%, totalling 8841 participants. Non-response (n = 5964) was largely due to refusals (61%), not completing the full survey (21%) or partial or incomplete information (i.e. participants not answering all questions which apply to them) (12%) [14]. A follow up study of NSMHWB participation confirmed that non-response was small at the aggregate level [14]. Data were provided by the Australian Bureau of Statistics (ABS) from a Confidentialised Unit Record File. This dataset is openly available to research institutions via the ABS.

### Data collection instruments

#### Depression and CVD

Respondents with depression in the last 12 months were identified using the Composite International Diagnostic Interview (CIDI 3.0), one of the most widely-used, structured diagnostic interviews for psychiatric disorders in the world. CIDI 3.0 is primarily used for epidemiological research and has demonstrated sound validity and reliability for diagnosing depression; the inter-rater reliability for any depressive disorder has been shown to be high (kappa statistic of 0.95 [15]) and moderate to good concordance with the Structured Clinical Interview for DSM-IV (SCID) has been observed [16]. Diagnostically, MDD is characterized by the presence of severely depressed mood persisting for at least two weeks [17]. Respondents were identified as having CVD on the basis of their response to the question 'have you had or been treated for a CVD condition (e.g. heart attack, angina, high blood pressure) over the past 12 months?' (research has shown a reasonable correlation between self-reported chronic diseases, such as diabetes, heart disease and asthma and those identified in medical records [18]). This process allowed us to classify people as those (1) without MDD or CVD, (2) with MDD but not CVD, (3) with CVD but not MDD, (4) with both MDD and CVD. The time frame of 12 months was selected for each condition to best reflect participants' current disease status.

#### Work outcomes

Workforce status was assessed using a reduced set of questions from the ABS monthly Labour Force Survey. Individuals reporting participation in the workforce on a full (≥35 h per week) or part time (less than 35 h per week) basis [13] were categorised as employed, versus those not participating in the labour force at the time of survey completion. Number of hours usually worked by employed participants in one week was recorded. Workplace absenteeism over the past month was assessed using the 'days out of role' item [13] (used in previous NSMHWB surveys, e.g. Lim et al. [19]). This entailed participants nominating how many days they were totally unable to work because of their health and if less than 30 days, the number of days they had to cut down on what was done or did not get as much done as usual because of their health. Work functioning was measured using an item on the World Health Organization Disability Assessment Schedule (WHO-DAS) where participants rated their difficulty with day-to-day work as none, mild, moderate, severe or extreme.

#### Co-variates

Demographic information included age, sex, registered marital status, area socioeconomic disadvantage (Decile 1-10; where 1 = most disadvantage and 10 = least disadvantage) [13], country of birth, main language spoken at home (English, other), physical activity in the past week (number of occasions spent walking for recreation, exercise, etc.), rurality (residing in major urban, other urban, other), education (dichotomised into pre and post-graduate attainment), body mass index (BMI) (calculated using the standard equation of weight divided by height squared [20]), psychological distress (Kessler-10) [21], social support (frequency of contact with family and friends) and current smoking status [13]. Participants self-rated their current mental and physical health using validated 5-point scales (excellent to poor), considered valid for measuring general health [22].

### Data analysis

Estimates and standard errors (SE) were derived using a complex estimation procedure to account for the stratified multistage survey design, oversampling and non-response [13], using the Jack-knife delete-2 technique. Probability (sampling) weights were applied to weight the sample back to the population from which the sample was drawn. Re-running the analysis in the same way without weights indicated similar odds ratios to the reported results (data not shown). No age limits were placed upon participant's inclusion, as n = 222 participants under the age of 18 years were reported to be in the workforce, as well as n = 197 participants over the age of 65 years. For some categorical variables, we retained the additional group of "no response or not applicable" where necessary, to include all respondents in our analyses. Due to a limited number of cases, the work functioning variable was dichotomised into *no difficulty *and *mild to extreme difficulty*. The days out of role variable (workplace absenteeism) was treated as a categorical variable. Previous studies have classified individuals reporting ≥5 days of missed work in the prior month as having significant work disability [23], therefore the following categories were applied: 0 days absent per month; 1-4 days absent per month; 5 days absent per month or more (linear regression modelling was not applied because data were not normally distributed).

A multivariate, logistic regression analysis was performed with workforce participation as the dependent variable, to explore differences in workforce participation according to MDD/CVD disease group, using methods described by Hosmer and Lemeshow (2000)[24]. Post-estimation tests were conducted to assess goodness-of-fit and specificity of the final model. This regression modelling strategy was also used to explore the relationship between disease group and work functioning. To assess the relationship between disease status and absenteeism, ordinal logistic regression was applied using the proportional odds model (Adjusted Wald statistic); the assumptions of which were met. Only participants indicating that they were participating in the workforce were included in the models assessing work functioning and absenteeism. Synergistic effects of CVD and MDD were assessed by the addition of a CVD/MDD interaction to a model containing separate main effects terms: CVD over the past 12 months (yes/no) and MDD over the past 12 months (yes/no). All measures of magnitude were presented as adjusted Odds Ratios (OR) with Jack-knife SEs and 95% confidence intervals (CIs). Stata 11 (survey procedures) was used for all statistical analyses. STROBE guidelines [25] were applied for the reporting of cross-sectional studies.

## Results

Table 1 displays the distribution of participants across disease groups, by workforce participation status and hours worked per week. The key characteristics of each group are displayed in Table 2. Those with MDD had the youngest mean age (36.7 years) while those with CVD only had the oldest mean age (62.1 years). Those with co-morbid MDD and CVD comprised the lowest proportion of males, followed by the MDD group. The co-morbid MDD and CVD group also reported: lowest proportion of excellent to good self rated physical and mental health and lowest frequency of physical activity over the previous week, the lowest proportion of participants with a normal BMI, the highest proportion of participants in lower socio-economic deciles and who exhibited moderate to high psychological distress. Those with MDD only comprised the highest proportion of smokers and non-married participants, and individuals with a normal BMI range. This group reported the highest frequency of physical activity in the previous week (Table 2). While the gender distribution for the prevalence of depression was relatively equal across the MDD only and MDD/CVD groups, there was a slightly greater proportion of women reporting MDD. This is consistent with the existing literature suggesting that affective disorders are more common in women than men [26].

**Table 1:** Number and proportion of NSMHWB survey participants, by disease group

	All participants (n = 8841)		Employed participants (n = 5499)		Working ≥ 35 hours per week (n = 3,499)	
Condition type	n	%	n	%	n	%

Neither CVD nor depression	6,079	68.8	4067	74.0	2,617	74.8

Depression only^+^	1,326	15.0	909	16.5	564	16.1

CVD only±	1,223	13.8	434	7.9	266	7.6

Co-morbid depression^+ ^and CVD±	213	2.4	89	1.6	52	1.5

Total	8841	100	5,499	100	3499	100

+Major depressive disorder (past 12 months); ^± ^Been told or treated for heart/circulatory condition (angina, heart attack, high blood pressure) in past 12 months

**Table 2:** Key characteristics of survey participants, by disease group (n = 8841)

	(1) Neither MDD nor CVD	(2) MDD only	(3) CVD only	(4) Co-morbid MDD & CVD
	
	n = 6,079	n = 1326	n = 1,223	n = 213
	Mean/Percentage	Mean/Percentage	Mean/Percentage	Mean/Percentage
	
	(95% CI)	(95% CI)	(95% CI)	(95% CI)
Age	42.5 (42.2, 42.8)	36.7 (35.7, 37.7)	62.1 (60.9, 63.2)	54.6 (52.1, 57.2)

Sex (male)	50.6 (49.7, 51.6)	45.4 (41.4, 49.4)	51.0 (47.5, 54.4)	40.9 (30.3, 51.5)

Country of birth (Australia)	71.2 (69.3, 73.2)	80.9 (77.6, 84.1)	71.6 (66.6, 76.6)	75.0 (66.1, 83.9)
Main language spoken at home (English)	90.4 (89.1, 91.6)	94.2 (91.6, 96.7)	92.7 (89.4, 95.9)	96.4 (92.5, 100.0)
Registered marital status (not married/single)	46.5 ( 45.0, 48.0)	64.7 (60.1, 69.3)	28.7 (25.0, 32.5)	41.2 (31.63, 50.7)

Post graduate education (yes)	57.0 (55.6, 58.4)	51.9 (48.3, 55.6)	46.8 (42.5, 51.1)	48.4 (37.7, 59.1)

Level of Area social economic disadvantage (Decile 1-5)+	44.7 (42.5, 46.9)	46.7 (42.3, 51.2)	49.15 (44.3, 54.0)	57.5 (46.3, 68.6)

Self-rated physical health (Excellent-Good)	89.8 (88.7, 90.8)	77.5 (74.8, 80.2)	76.2 (72.6, 79.8)	44.4 (33.5, 55.2)

Self-rated mental health ± (Excellent-Good)	96.1 (95.5, 96.8)	72.7 (68.0, 77.3)	92.4 ( 90.2, 94.5)	53.5 (52.8, 74.2)
Psychological distress (Moderate to high distress)	20.7 (19.2, 22.1)	64.3 (60.7, 67.9)	24.1 (19.8, 28.3)	68.9 (56.9, 81.0)

Smoke (yes)	20.6 (19.1, 22.2)	38.5 (34.4, 42.6)	10.6 (8.1,13.0)	28.0 (16.1, 39.9)

Body Mass Index (% normal)	44.5 (42.6, 46.3)	49.3 (45.5, 53.1)	23.9 (20.1, 27.6)	18.9 (9.6, 28.3)

+Frequency of physical activity in past week	5.2 (4.9, 5.5)	5.6 (4.9, 6.2)	4.3 (3.6, 4.9)	4.2 (2.7, 5.7)

+Most disadvantaged; does ± not include the full 8841 participants due to missing data

### Relationship between disease status and workforce participation

Multivariate logistic regression, adjusting for sex, age, marital status, rurality, smoking, area social disadvantage, education, country of birth, main language spoken, self-rated physical health and social support, revealed that individuals with co-morbid MDD and CVD were approximately half as likely to be working compared with those without either condition (adj OR 0.4, 95% CI: 0.3-0.6) (Table 3). Those with MDD only (adj OR: 0.8, 95% CI: 0.7-0.9) and CVD only (adj OR: 0.8, 95% CI: 0.6-0.9) also reported significantly reduced odds of participation. Since age is related to both work participation and disease status, we conducted a sensitivity analysis that stratified by age group. We selected the following age groups on which to base our analysis: under 36 years (the lowest mean age of the 4 groups (MDD only)), 36-65 years (retirement age in Australia at the time of survey), and over 65 years. When we explored the odds of reduced participation for those aged between 36 and 65 years, similar odds ratios were observed; those with co-morbid CVD and MDD reported reduced odds of participation (adj OR: 0.6, 95% CI: 0.4-0.8). However, for those aged under 36 years, the odds of work participation for those with co-morbid CVD and MDD was more pronounced (adj OR: 0.2, 95% CI: 0.1-0.9). No significant effects were observed between disease group and work participation for those over 65 years of age (data not shown).

**Table 3:** Logistic regression model for the relationship between workforce participation and disease group (n = 8841)

**Employment status**^**+**^	Unadjusted	Adjusted	Jack-knife	Confidence intervals
	Odds ratio	Odds ratio±	Standard error	(95%)
Condition				

Neither CVD nor MDD	1.0			

MDD only	1.0	0.8*	0.1	0.7, 0.9

CVD only	0.3*	0.8*	0.1	0.6, 0.9

Co-morbid MDD and CVD	0.3*	0.4*	0.1	0.3, 0.6

CVD-MDD interaction		0.7	0.3	0.4, 1.5

^+ ^0 = Not employed in workforce 1 = full or part-time employment in the workforce, where 0 is the reference group; ± = Adjusted for sex, age, marital status, rurality, smoking, area social disadvantage, education, country of birth, main language spoken, self rated physical health and social support* = *p *< 0.05; Goodness of fit [27] and link tests produced non-significant test statistics (*p *= 0.10 and *p *= 0.29 respectively), reflecting goodness of fit and sound model specificity

### Relationship between disease status and work functioning

Of all the groups, employed individuals with co-morbid CVD and MDD were most likely to experience mild to extreme impairments in work functioning (Table 4). Compared with the healthy reference group, this group was 8 times more likely to report impaired functioning (adj OR: 8.1, 95% CI: 3.8-17.3), followed by those with MDD alone (adj OR 3.8, 95% CI: 3.0-4.8), after adjusting for age, sex, country of birth, education, smoking, chronic lifetime physical condition, number of hours worked per week. Compared with the healthy reference group, those with CVD only reported no significant differences in work functioning. Because functioning could be associated with time spent at work, we further stratified work functioning by hours usually worked per week. Those with co-morbid MDD and CVD, again, reported greater odds of poor functioning; those working on a full time basis reported greatest odds (Table 5) (large CIs reflect small number of cases).

**Table 4:** Logistic regression model for the relationship between impaired work functioning and disease group (employed participants) (n = 5499)

**Amount of difficulty in day to day work**^+^	Unadjusted	Adjusted	Jack-knife	95%
	Odds Ratio	Odds ratio±	Standard	Confidence
			Error	intervals
Condition				

Neither CVD nor MDD	1.0			

MDD only	4.3*	3.8*	0.4	3.0, 4.8

CVD only	1.0	0.9	0.2	0.6, 1.4

Co-morbid MDD & CVD	10.7*	8.1*	3.0	3.8, 17.3

CVD-MDD interaction		2.4*	1.0	1.01, 5.7

^+ ^0 = None or not known, 1 = Mild- Extreme difficulty, where 0 is the reference group; ± = Adjusted for age, sex, country of birth, education, smoking, chronic lifetime physical condition, number of hours worked per week; Goodness of fit [27] and link tests produced non-significant test statistics (*p *= 0.24 and *p *= 0.051 respectively), reflecting goodness of fit and sound model specificity

**Table 5:** Logistic regression model for the relationship between impaired work functioning and disease group, by hours worked (n = 5499)

**Amount of difficulty in day to day work**^**+**^	Unadjusted	Adjusted	Jack-knife	Confidence
	OR	OR ±	Standard Error	intervals
*< 35 hours per week*				
Neither CVD nor MDD	1.0	1.0		

MDD only	3.1*	3.4*	0.64	2.3, 5.0

CVD only	0.8	0.8	0.3	0.3, 1.6

Co-morbid MDD & CVD	5.9*	4.5*	2.2	1.7, 11.8

≥*35 hours per week*				

MDD only	4.6*	4.0*	0.6	2.9, 5.6

CVD only	1.1	0.9	0.3	0.5, 1.7

Co-morbid MDD & CVD	14.9*	10.6*	5.3	3.9, 29.0

± = Adjusted for age, sex, country of birth, education, smoking, chronic lifetime physical condition

### Relationship between disease status and workplace absenteeism

After adjustments for age, sex, marital status, education, smoking, mental and physical self-rated health and area social disadvantage, those with co-morbid CVD and MDD were three times more likely to belong to a higher category of days absent from work (adj. OR: 3.0, 95% CI: 1.4-6.6) (Table 6). Those with MDD were also significantly more likely to report a higher category of workplace absenteeism (adj. OR: 1.8, 95% CI: 1.4-2.4). Those with CVD only reported no differences in workplace absenteeism compared with the healthy reference group. Further, we stratified workplace absenteeism by hours usually worked per week (Table 7). Those with co-morbid MDD and CVD reported greatest odds of workplace absenteeism for both part time workers (employed less than 35 h per week) (adj OR 3.6, 95% CI: 1.4-11.7) and full time workers (employed ≥35 h per week) (adj OR: 3.0, 95% CI: 1.3-8.0). Those with MDD only also had increased odds of workplace absenteeism, compared with the healthy reference group for both part time (adj OR: 1.9, 95% CI: 1.3-2.9) and full time (adj OR: 1.7, 95% CI: 1.2-2.5) workers.

**Table 6:** Ordinal logistic regression model for the relationship between workplace absenteeism and disease group (employed participants) (n = 5499)

**Category of days unable to work**^**+**^	Unadjusted	Adjusted	Jack-knife	Confidence
	OR	**OR±**	Standard Error	intervals 95%
Condition				
Neither CVD nor MDD	1.0	1.0		

MDD only	2.7*	1.8*	0.2	1.4, 2.4

CVD only	1.0	1.0	0.2	0.6, 1.6

Co-morbid MDD & CVD	4.5*	3.0*	1.2	1.4, 6.6

CVD-MDD interaction		1.8	0.8	0.7, 4.6

+ 0 = 0 days per month (reference group), 1 = 1-4 days; 2 = 5 days per month or more; ± = Adjusted for: age, sex, marital status, education, smoking, area social disadvantage, self rated mental and physical health *p < 0.05; Post-estimation tests revealed good model specificity (linktest *p *= 0.45), and no violation of proportion odds assumption (*p *= 0.36)

**Table 7:** Ordinal logistic regression model for the relationship between workplace absenteeism and disease group, by hours worked (n = 5499)

**Number of days unable to work**^**+**^	Unadjusted	Adjusted	Jack-knife	Confidence
	OR	OR ±	Standard Error	intervals
*< 35 hours per week*				
Neither CVD nor MDD	1.0	1.0		

MDD only	2.5*	1.9*	0.4	1.3, 2.9

CVD only	1.0	1.1	0.6	0.4, 3.3

Co-morbid MDD & CVD	5.0*	3.6*	2.1	1.4, 11.7

≥*35 hours per week*				

MDD only	2.2*	1.7*	0.3	1.2, 2.5

CVD only	0.7	0.9	0.2	0.5, 1.6

Co-morbid MDD & CVD	3.2*	3.0*	1.5	1.3, 8.0

+ 0 = 0 days per month (reference group), 1 = 1-4 days; 2 = 5 days per month or more; ± = Adjusted for: age, sex, marital status, education, smoking, area social disadvantage, self rated mental and physical self rated health *p < 0.05; Post-estimation tests revealed good model specificity

In addition, we ran all of the models with disease status coded to include those with lifetime CVD and MDD, in order to assess the association between long term disease status and work outcomes. These analyses yielded similar odds ratios for all outcomes (data not shown).

Finally, we explored the interactive effects of MDD and CVD on all three work outcomes. We found a significant interaction between MDD and CVD on work functioning (*p *= 0.04). The effects of MDD and CVD on workforce participation and absenteeism (for both part and full time workers) were shown to be additive rather than synergistic; interaction terms were non-significant (*p *> 0.05).

## Discussion

Our research findings demonstrate that major depression which co-occurs with CVD is associated with poor work outcomes, including reduced workforce participation and greater work functioning impairments and workplace absenteeism. For all outcomes, those with co-morbid CVD and MDD experienced greater impairment than those with either condition by itself. While no significant interactive effects were found between MDD and CVD on work participation or absenteeism, a synergistic relationship was observed between MDD and CVD on workforce functioning, indicating that the combined effect of these conditions on functioning is greater than the sum of the effects of depression and CVD when they occur independently. To our knowledge, this is the first time the burden of, and interaction between MDD and CVD, specifically, has been explored on work outcomes at the population level.

Our findings are consistent with cross-sectional studies conducted in Europe [10], Northern America [23] and Australia [9] in which other co-morbid populations have also demonstrated poorer work outcomes. For example, Baune (2007) found that MDD co-occurring with any medical disorder was strongly associated with lower full-time working status and significantly more disability days [10]. Further, our findings add to others in this field, by confirming a synergistic effect of co-morbid MDD and chronic physical conditions on functioning [12], but not work absenteeism [11].

The synergistic relationship observed between MDD and CVD on work functioning, but not participation or absenteeism, suggests the negative effects of this co-morbidity are most pronounced for functional outcomes. Previous studies in MDD and diabetes populations [12] also support this finding. It would be expected that depression impacts mental functioning and CVD impacts physical functioning, and that cumulatively, the conditions combine to impede overall functioning. However, the interaction we observed between MDD and CVD on functioning may be a result of depressive symptoms exacerbating perceived impairment due to CVD, or may reflect greater physical symptom severity which can impede mental and physical components of functioning; essential for work productivity. That is, those who are depressed may have more severe forms of the disease. Further research is required to disentangle the association between this co-morbidity and mental and physical functioning.

There are several explanations for our finding of poorer work outcomes in those with co-morbid MDD and CVD. While its cross-sectional design precludes us from making causal inferences about the association between co-morbid mental and physical conditions and workforce status, we speculate that employment status may be influenced by both internal and external factors. As depression is a recognised risk factor for CVD [28] and stress is a shared risk factor for depression and CVD [29], stress may, in fact, act as a mediator in this relationship. Alternatively, risk factor clustering could exacerbate the effects of both CVD and MDD. For example, individuals with MDD may be more likely to report alcohol and tobacco use [30] and poor dietary regimes [31] and physical activity levels [32]; many of which occur simultaneously. Indeed, these behaviours can impede recovery after a CVD event, increase the risk of cardiac events and contribute to the physiology which underlies disease progression.

Moreover, we observed significant age-related effects of this co-morbidity on workforce participation; those under 36 years reported more pronounced reductions in participation than those aged 36-65 years, and no significant reductions were observed for those over 65 years. There are several possible explanations for this finding. For example, individuals who have experienced this co-morbidity at a young age may have: more chronic symptoms with greater severity, greater difficulty managing their conditions due to competing interests (such a child rearing), or different disease management or treatment plans compared with their older counterparts. Further, since depression can manifest either before or after CVD onset, and order of onset has been shown to result in differential outcomes [33], it is possible that the clinical course of MDD and/or CVD and their associated outcomes, differs in younger persons compared with older individuals.

This study has the following strengths. Compared with most other existing studies [34], our study used a valid psychiatric diagnostic instrument to assess MDD. While a diagnostic interview is time consuming, it is a more accurate method for the classification of depression than self-report methods. Another strength of our study is its robustness due to the use of a large probability sample from the general population. However, some study limitations should also be acknowledged. Self-report measures were used to define participants' CVD status which may have led to recall bias, misclassification or incorrect identification of CVD. This may have resulted in an under-reporting of CVD and thus a possible dilution of the CVD effect. Similarly, it is possible that MDD may have also been under-reported; a study of NSMHWB non-responders revealed non-response may be associated with mental illness for younger individuals and males [14]. However, the representativeness of this sample has been reported previously [14]. A further limitation of the study is the large CIs and SEs resulting from small numbers of employed participants with both co-morbid depression and CVD.

More research is needed to further understand the inter-relationships and the implications for developing effective prevention and intervention programs for people with co-morbid CVD and MDD. Longitudinal cohort studies have the potential to reveal both the long-term and causal impact of depression and CVD on workforce retention, early retirement and disability, as observed in international studies [35]. Future longitudinal studies should investigate whether this trend is comparable for individuals with co-morbid MDD and CVD. Further, randomised controlled trials that aim to improve vocational outcomes of individuals with co-morbid depression and CVD are required. To date, existing trials in this area have focused more on clinical outcomes, over psychosocial or functional outcomes such as employment. Several of these trials have, however, demonstrated positive effects of depression management on mental health functioning in those with CVD [36]. While it is likely that these benefits extend to vocational functioning, there is limited evidence to support this. Several studies in this area are currently exploring the impact of depression management after a cardiac event on work outcomes [36,37]. As it is likely that the relationship between disease and employment status is bi-directional, interventions could be of a work-based nature, where occupational programs have the potential to improve disease management, or alternatively, of a psychological nature, where treating depression is likely to enhance both work and psychological outcomes in those with CVD.

## Conclusions

It has been argued that the occupational rehabilitation needs of people with co-morbid depression and chronic conditions are currently being underestimated [38]. Our findings highlight that those exhibiting co-morbid MDD and CVD are at high risk of functional impairments and work absenteeism. This should be considered by the relevant health professionals working in this field (psychologists, occupational therapists, cardiologists and rehabilitation nurses). The implementation and evaluation of targeted interventions in this population which facilitate work resumption, retention and productivity have the potential to be individually, organisationally and economically advantageous. Given the emergence of co-morbid psychological distress and chronic disease as a growing public health issue affecting workers in Western economies [39], mental and physical medical co-morbidities need to be prioritised given their prevalence and subsequent burden.

## Abbreviations

CVD: Cardiovascular disease; MDD: Major depressive disorder; CHD: Coronary heart disease; NSMHWB: National survey of mental health and wellbeing; ABS: Australian bureau of statistics; CIDI 3.0: Composite international diagnostic interview 3.0; SCID: Structured clinical interview for DSM-IV; BMI: Body mass index; NHPA: National health priority areas; WHO-DAS: World health organization disability assessment schedule; SE: Standard errors; OR: Odds ratios; CI: Confidence intervals; STROBE: Strengthening the reporting of observational studies in epidemiology

## Competing interests

The authors declare that they have no competing interests.

## Authors' contributions

AO conceptualised the paper, analysed and interpreted data, and wrote the original version of the manuscript. EDW assisted with statistical analysis and interpretation of the data, and contributed to drafts of the manuscript. CEW assisted with statistical analysis and interpretation of the data, and contributed to drafts of the manuscript. BO critically revised the manuscript. KS assisted with conceptualising the paper, statistical analysis and interpretation of the data, and critically revised drafts of the manuscript. All authors read and approved the final version of the manuscript.

## Pre-publication history

The pre-publication history for this paper can be accessed here:

http://www.biomedcentral.com/1471-2458/12/47/prepub
